# Visualization of synaptic domains in the Drosophila brain by magnetic resonance microscopy at 10 micron isotropic resolution

**DOI:** 10.1038/srep08920

**Published:** 2015-03-10

**Authors:** Choong H. Lee, Stephen J. Blackband, Pedro Fernandez-Funez

**Affiliations:** 1 Department of Neuroscience, McKnight Brain Institute, University of Florida, Gainesville, FL. 32611, USA; 2 National High Magnetic Field Laboratory, University of Florida, Gainesville, FL. 32611, USA; 3 Department of Neurology, Genetics Institute, and Center for Translational Research on Neurodegenerative Diseases, University of Florida, Gainesville, FL 32611, USA

## Abstract

Understanding the complex architecture, connectivity, and pathology of the human brain is a major application of magnetic resonance imaging (MRI). However, the cellular basis of MR signal is still poorly understood. The advent of MR microscopy (MRM) enables imaging biological samples at cellular resolution, helping to interpret the nature of MR signal at the cellular level. In this regard, the small *Drosophila* brain can reveal key aspects of MR signal through the visualization of complex, intact neuronal structures in their native spatial arrangement. Applying state-of-the-art MR technology, we imaged fixed *Drosophila* heads at 10 μm isotropic resolution by two endogenously contrasted MRM sequences. The improved MRM sensitivity described here delivered the highest 3D resolution of an intact animal head reported so far. 3D fast low angle shot (FLASH) revealed strong signal in most internal tissues, particularly in the brain cortex, which contains the cell bodies of neurons and glia. Remarkably, 3D diffusion weighted imaging (DWI) delivered unprecedented contrast within the modular brain neuropil, revealing hyperintense signal in synapse-rich microdomains. Thus, the complex *Drosophila* brain revealed unknown features of FLASH and DWI with potential applications in characterizing the structure and pathology of the mammalian brain.

In the last few years, we have witnessed a renaissance in microscopy, with significant progress towards mesoscale resolution in light microscopy, cellular resolution in living animals, and tissue resolution for non-invasive technologies^1^,^2^. Examining tissue structure and function non-invasively is one of the great outstanding scientific challenges of our time. Non-invasive imaging enables the analysis of complex structures such as the brain in their natural environment and, thus, has many clinical and basic research applications. Magnetic resonance imaging (MRI) is the gold standard for non-invasive imaging modalities since its inception in the 1970s^3^,^4^,^5^ because it provides three-dimensional (3D) information of soft tissues from water content, thus avoiding harmful radiation (Fig. 1a). The inception of MR microscopy (MRM) in 1986 by three independent groups demonstrated the potential of MR to probe the biological architecture at the cellular level^4^,^5^,^6^. Continuous advances in magnet strength, strong/fast-switching gradients, and radio-frequency (RF) detector sensitivity have improved image resolution to 100-5 μm, with new applications from animal research^7^ (Fig. 1b) to cellular architecture^8^,^9^,^10^.

Since the size of the RF detector is inversely proportional to its sensitivity, small specimens such as excised brain fragments can be imaged at higher resolution than intact brains. However, intact heads enable examining the complex cellular composition, architecture, and connectivity of the complete brain. Here, the small insect head can contribute to the refinement of MRM technology and the understanding the nature of the MR signal in complete and complex brains. In the early 2000s, the large heads of blowflies and honeybees (3–5 mm wide) and moths (8 mm wide) were imaged by MRM at 15–50 μm in-plane resolution^11^,^12^,^13^. Despite their size, imaging of living blowflies and moths by MRM revealed few anatomical details of their brains^11^,^13^. MRM of the fixed honeybee head revealed the modular organization of the brain neuropil, including the massive mushroom bodies as well as the three synaptic domains of the optic lobes^12^. However, bees are not genetically tractable and, thus, have limited application for manipulating brain structure and physiology.

For decades, the fruit fly *Drosophila melanogaster* has proved a powerful model system for the genetic dissection of many developmental processes and has contributed to uncovering the genetic and cellular mechanisms involved in neuronal specification and function^14^. The introduction of novel genetic tools for neuronal tracing over the last decade has identified neuronal circuits in the *Drosophila* brain, which can be mapped in a virtual brain^15^,^16^,^17^. *Drosophila* is an ideal tool for advancing non-invasive imaging techniques because it combines a small size (600 × 300 × 150 μm) with high complexity (10^5^ neurons, 10^7^ synapses). The organization of the *Drosophila* brain is relatively well-known from histological and fluorescence studies, with the neuronal and glial cell bodies on the outer rim (the cortex) surrounding a modular neuropil with several synaptic domains (see Fig. 2)^18^. Many of these microdomains have been characterized functionally, with roles in olfaction and taste (antennal lobe, suboesophageal ganglion), memory and higher order processing (mushroom bodies, lateral horn), locomotion (central complex), and visual processing (medulla, lamina, and lobula plate). Recent advances in ultramicroscopy, optical projection tomography, and X-ray computed tomography attempted to image complete *Drosophila* bodies non-invasively, but neither technique produced enough resolution to visualize the brain's modular architecture^19^,^20^,^21^. A main advantage of the harmless MRM is that it can generate contrast by detecting water content and local differences in tissue architecture and composition. The first application of MRM in *Drosophila* imaged living pupae on an 18.8 Tesla (T) magnet, which achieved 19.5 μm isotropic resolution (μm^3^) using 3D fast low-angle shot (FLASH) and 12.5 μm in-plane by 2D rapid acquisition with refocused echoes (RARE)^22^. However, this study revealed no details of the internal brain anatomy. A recent paper reported a 10 × 10 × 80 μm resolution using 2D FLASH MRM and 20 μm^3^ by 3D FLASH in a 9.4 T magnet with a stronger gradient (950 mT/m) in anesthetized adult flies^23^. This approach generated low contrast in the central brain, but detected the progressive degeneration of the large flight muscles of the thorax.

Here, we present 3D MRM of complete, fixed *Drosophila* heads at 10 μm^3^, the highest resolution reported so far of an intact animal head. We used state-of-the-art hardware that included a 1 mm planar RF microcoil (Fig. 1c), a strong and fast-switching planar gradient (1.1 T/m/A), and a 600 MHz (14.1 T) vertical-bore magnet (Fig. 3). We imaged the same *Drosophila* heads by two MRM sequences - 3D FLASH MRM and 3D diffusion-weighted imaging (DWI) – using endogenous contrast, resulting in unprecedented detail of the complex microarchitecture of the brain neuropil. Although FLASH MRM revealed strong signal of most head structures, DWI provided extraordinary contrast of the modular neuropil, allowing the identification of several known brain centers enriched in synaptic connections. These studies underscore the ability of MRM to non-invasively describe the underlying architecture of complex biological specimens at high resolution and suggest that the small *Drosophila* brain can contribute to better interpreting the nature of the MRM signal at the tissue and cellular scales.

## Results

### Imaging the small, modular Drosophila brain

We first prepared two brains for traditional imaging - fluorescence microscopy and histological sections - to provide anatomic support for MRM imaging (Fig. 2). The fluorescent brain expressed a Synaptobrevin (Syb)-GFP reporter fusion pan-neurally to label the microarchitecture of the neuropil. We also labeled the neuronal nuclei with the anti-Elav antibody. We imaged these brains at low magnification (100×) to visualize the whole brain (Fig. 2A). The fluorescent images illustrate the cortical position of the neuronal cell bodies around the central brain and the optic lobes (Fig. 2Ag, magenta). The internal brain architecture consists of several recognizable synaptic domains in the central brain as well as the three synaptic neuropils of the optic lobe (Fig. 2A, green). We also imaged 1 μm thick histological sections of the whole head, which preserves the natural distribution of all head structures (Fig. 2B). The cell bodies in the cortex appeared in a lighter blue surrounding the neuropil, which discriminates between dense synaptic regions (darker) and nerves (lighter). The head sections also include internal structures removed during brain dissection: the modular retina and lamina, head muscles (dark blue), the ocelli (single eyes), fat bodies (dark green), and trachea, the respiratory organ of invertebrates (light vacuoles). We have highlighted the main areas of interest in the optical brain images (Fig. 2) as reference for the MR microimages (Figs. 4 and 5).

### MRM hardware and selection of imaging sequences

For this paper, we employed the state-of-the-art MRM hardware used previously for visualizing single neurons from excised fragments of human brain to image a complete insect head for the first time. The basic hardware includes a powerful 600 MHz (14.1 T) magnet and a strong and fast-switching planar gradient with a maximum amplitude of 66 T/m (Fig. 3), almost two orders of magnitude larger than the gradient generated in a recent paper imaging whole *Drosophila* bodies^23^. A key element of the hardware is the RF coil. Traditionally, solenoid coils have been employed for high-resolution MR to exploit their field homogeneity. Instead, we used RF micro surface coils that maximize compatibility with the small and planar set of gradient coils, which is the basis of their great strength and switching power. The RF coil is a flat insert into the gradients designed as interchangeable surface coils (Figs. 3a–d). These surface coils increase signal-to-noise ratio (SNR), but also allow easy sample access, placement, and correlative histology. This powerful arrangement has facilitated the first direct imaging of single mammalian cells^10^ and cellular level fiber tract mapping^24^.

**Figure 1:**
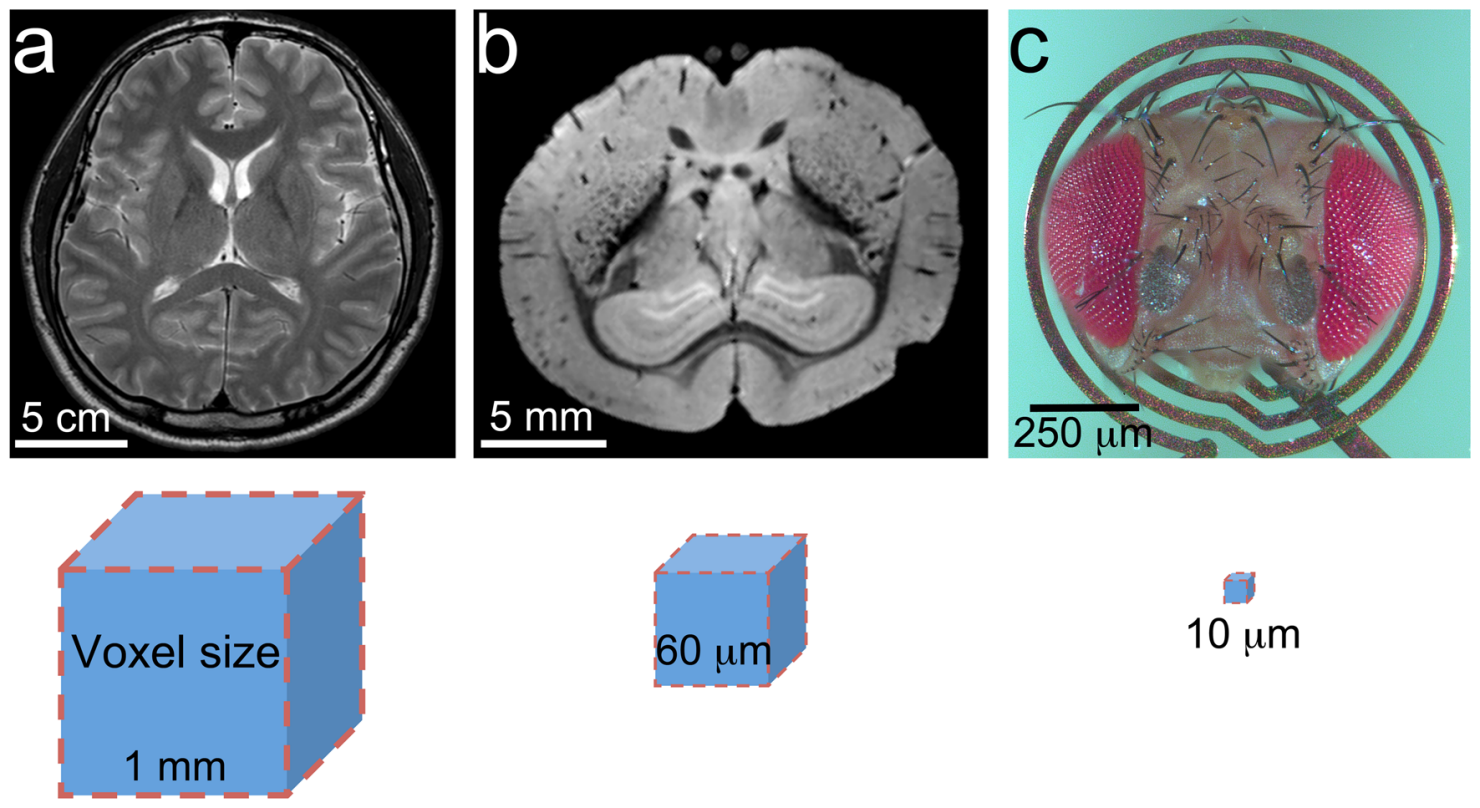
Illustration providing a general representation of the scales of MR imaging between mammalian and fly brains. (a) Human brain imaged at 1 mm resolution as indicated by voxel size. (b) Mouse brain imaged at 60 μm. (c) *Drosophila* head on an RF microcoil prepared for imaging at 10 μm. Voxel sizes not shown at scale.

One of the advantages of MR is the ability to utilize multiple imaging sequences to extract structural, connectivity, and functional information from the tissue. For our structural studies, we chose two very different sequences, FLASH and DWI. 3D FLASH imaging is a T1-weighted technique mostly applied for real-time and functional diagnostics due to its fast acquisition time^25^. DWI is a newer MR contrast mechanism that has found many clinical applications for detecting physiological and pathological states of the brain, specifically for identifying areas affected by stroke^26^,^27^,^28^. DWI relies on detecting water diffusion per voxel, producing contrast based on tissue structures that affect water diffusion. We found recently that, at less than 10 μm^3^ resolution, water diffusion dynamics distinguished the cell body (negative signal) from mammalian neurons in excised brain fragments^10^. The use of a strong gradient (3,000 mT/m) avoided resolution limitations, like free water diffusion, inherent in high resolution MRM. We hypothesized that similar anatomic detail could be obtained from a whole fly head using even stronger/fast-switching gradient coils (1.1 T/m/A)^29^ and optimized RF 1 mm external diameter microcoils that fit the complete *Drosophila* head (Fig. 1c). The advantage of DWI is that many relevant parameters can be extracted from the sample by acquiring images in different directions or with different b-values, which can be useful for constructing fiber track mapping and diffusion maps, respectively. However, imaging in 10 μm^3^ voxels resulted in long acquisition times, a total of 80 h for combining FLASH and DWI images for one brain. Thus, we scanned the samples using one direction and one b-value (high) that produced high contrast while minimizing signal loss based on previous imaging experience. Since the T2-weighting was minimized by reducing the echo time to 4.7 ms, the MR contrast is generated mostly due to water diffusion through the brain.

**Figure 2:**
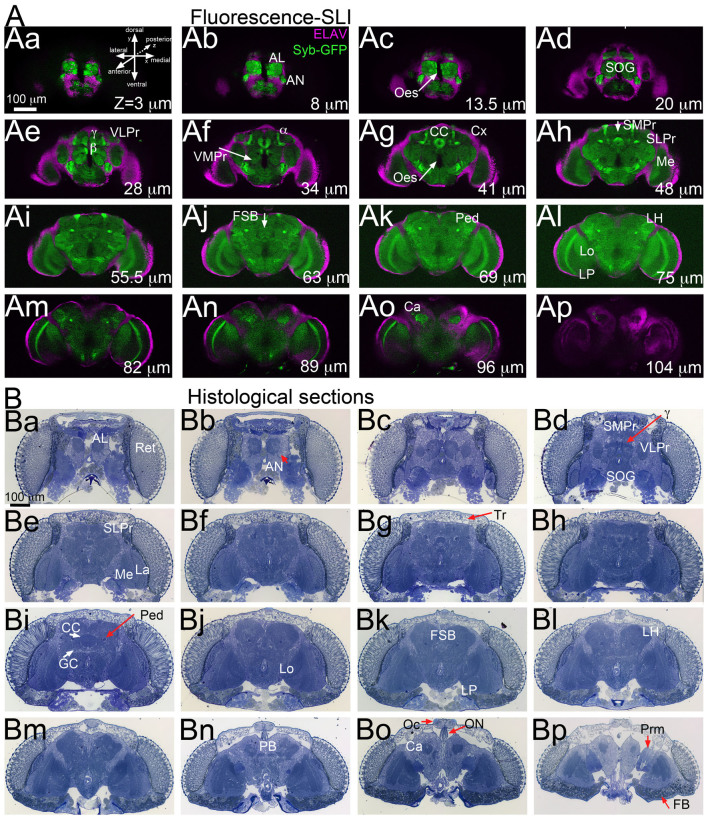
Drosophila brains imaged by optical microscopy. (A) Frontal optical sections of a fluorescent brain imaged by structured light imaging (SLI). Fly brains expressing synaptic-bound GFP (Syb-GFP), green in all neurons under the control of Elav-Gal4 display green signal throughout the neuropil. The signal of Syb-GFP enables the identification of several brain centers and axonal paths. The anti-Elav antibody (magenta) labels the nuclei of all neurons in the cortex, surrounding both the central brain and the optic lobes. (B) Frontal serial semi-thin sections (1 μm thick) of a whole head stained with toluidine blue. The intensity of the staining reflects the underlying organization of different brain regions, with the darker blue indicating compact axonal branches (ped) and synaptic microdomains (MB, EB). Depth of selected optical sections is indicated in μm, from anterior to posterior. α, β, γ: mushroom body lobes; AL: antennal lobe; AN: antennal nerve; Ca: calyx; CC: central complex; Cx: cortex; FB: fat bodies; FSB: fan-shaped body; GC: giant commissure; La: lamina; LH: lateral horn; Lo: lobula; Lp: lobula plate; Me: medulla; Oc: ocelli; Oes: oesophagus; ON: ocellar nerve; Ped: pedunculus; Prm: posterior retractor muscle; Ret: retina; SMPr; superior-medial protocerebrum; SLPr: superior-lateral protocerebrum; SOG: suboesophageal ganglion; Tr: trachea; VLPr: ventro-lateral protocerebrum; VMPr: ventro-medial protocerebrum. The orientation of all the brain series is indicated in in panel Aa.

We collected all 3D series, optical and MRM, along the frontal plane (transverse sectioning), from front to back. To compare optical and MRM brain series, they were all oriented following the convention for optical imaging, with the plane of imaging in the X- and Y-axes, and the depth represented by the Z-axis (see Fig. 2Aa). For MRM, the Z-axis is aligned with the direction of the external magnetic field or B_0_. For reference, the most anterior section containing the brain in each series was arbitrarily designated as the start point (Z = 0 μm) and the depth in microns indicates the position along the Z-axis.

### FLASH MRM detects internal structures within the fly head

2D and 3D FLASH MRM had been used before with whole *Drosophila* bodies because it provides optimum contrast between different structures within the same organ, as in the human brain, while limiting the acquisition time (1.5 hours)^22^,^23^. To produce high-resolution structural data at 10 μm^3^, we collected 3D FLASH MRM of fixed *Drosophila* heads with long acquisition times (36 h) due to the reduced water content in the small voxels. 3D FLASH recorded hyperintense signal from most internal head structures, including the brain, the retina, the lamina, and head muscles, and low signal from the surrounding fat bodies (Fig. 4). These images demonstrated high contrast between the retina, which contains the photoreceptors and support cells, and the lamina, which contains the axonal projections of the photoreceptors and their synaptic contacts with lamina neurons (Figs. 4a–d). The brain was bright but the outer rim and the boundary between the brain and the optic lobes was hyperintense at depths between 90–120 μm (Figs. 4j–l). The distribution of this hyperintense signal corresponds to the cortical location of cell bodies as seen by fluorescence and histological sections (Fig. 2). At a depth of 70–80 μm, a hypointense region likely corresponding to the giant commissure interneurons crossing the brain right above the esophagus (Figs. 4h–i). This small group of large axons is also visible in the histological sections (Fig. 2Bi). The pair of posterior retractor muscles behind the brain also generated signal in FLASH (100–120 μm deep), although the signal was weaker than in the brain (Figs. 4k–m). Despite the strong signal, FLASH revealed little contrast inside the brain, which hindered the identification of the modular architecture of the neuropil (Figs. 4i–l). Overall, 3D FLASH at 10 μm^3^ detected most internal head structures and differentiated the retina from the lamina, two close visual structures. The most salient aspects of these images are the hyperintense signal of the cortex and hypointense signal of the giant commissure interneurons, suggesting that FLASH has high sensitivity for cell bodies and low for axons.

### DWI resolves synaptic neuropils with high contrast

In 3D DWI at 10 μm^3^, most of the signal originated in the brain and fat bodies, whereas the retina and the lamina were hypointense (Fig. 5). Remarkably, DW MRM produced high contrast in the neuropil, visually resolving several synaptic microdomains. From 10 to 30 μm deep, the antennal lobes and the suboesophageal ganglion were hyperintense compared to the surrounding tissue (Figs. 5b–d). Between 30–50 μm deep, a thin symmetrical hyperintense MR signal revealed the position of the vertical axonal terminals of the mushroom body lobes (Figs. 5d–f). At 60 to 110 μm deep, the axonal bundles of the mushroom body neurons (the peduncles) appeared as hypointense MR signal (Figs. 5h–k) even though their terminal projections (the mushroom body lobes) were hyperintense (Fig. 5f). In the central region of the brain, DWI detected the distinctive circular shape of the central complex at 80 μm deep (Fig. 5i). In the optic lobes, the medulla first emerged at 40 μm, the lobula at 90 μm medial to the medulla, and the lobula plate at 110 μm, between the medulla and the lobula (Figs. 5e, j, l). The three synaptic centers of the optic lobes were clearly delineated because the axon fibers connecting them appeared hypointense (Figs. 2Bm and 5m). As opposed to FLASH, the esophageal opening was hypointense in all images; thus, DWI detected a signal from the brain, but not from closely associated structures, such as the lamina and the esophagus. Interestingly, the same cortical areas occupied by cell bodies that were hyperintense by FLASH (Fig. 5k–m), were hypointense by DWI (Fig. 5k–m).

### 3D reconstruction of MR microimages

Since 3D FLASH and DWI displayed high contrast of the cortex and neuropil microdomains, we segmented these areas to obtain the first detailed 3D model of the *Drosophila* brain from an intact head by non-invasive imaging. 3D rendering of all the layers resulted in a model of the *Drosophila* brain similar to models derived from confocal imaging despite the lower resolution of the MR images (Fig. 6). Since the MRM brain model was acquired from whole heads, it contained additional structures typically removed during dissection for fluorescent microscopy: retina, lamina, and muscles (Fig. 6a–c and e). Since the FLASH and DWI sequences revealed different signal for the cortex, FLASH provided critical input to identify the cortical layer (Fig. 6a–c). Removing cortex, retina, and lamina, the MRM brain model appeared similar to the models created by confocal microscopy, except for the rough edges due to the relatively large MRM voxel size, i.e. the inherent limitation of resolving power of MRM even with the high spatial resolutions (10 μm iso-cubic voxel size) (Fig. 6d). The posterior view of the brain showed the close spatial connection of the lamina and the retina with the outer layer of the optic lobes, the medulla (Fig. 6e). A dorsal view showed the distribution of the cell bodies in a cross-section (Fig. 6f) and the position of the mushroom bodies behind the antennal lobes (Fig. 6g). Lastly, a series of slices showed internal brain centers: the antennal lobes, antennal nerve, and the suboesophageal ganglion (Fig. 6h); the mushroom bodies, the anterior protocerebrum, and the medulla (Fig. 6i); and the central complex, the lateral horn, the lobula, and the posterior protocerebrum (Fig. 6j). Overall, this 3D MRM model of the fly brain displayed several known domains with the expected spatial distribution.

### Tissue-specific FLASH and DWI signal

We next compared directly the selectivity and specificity of the images obtained with 3D FLASH and 3D DWI from the same slice position in the through-plane encoding direction. We first focused on the internal eye structures, the retina and the lamina. The retina consists of repeated visual units, the ommatidia, each composed of eight photoreceptors and several support cells (Fig. 7a, arrow). The lamina lies underneath the retina and maintains a similar modular architecture, keeping the axonal projections of the photoreceptors from the same ommatidia in highly ordered cartridges (Fig. 7a, arrowhead). In 3D FLASH microimages, the lamina appeared hyperintense, whereas the retina showed weaker signal (Fig. 7b). Since both structures produced homogeneous contrast, this resolution was insufficient to detect the repetitive columnar organization of the retina and lamina. As opposed to FLASH, both retina and lamina were hypointense in DWI (Fig. 7c), which could be due to the lower density of these structures compared to the brain neuropil, thus increasing water diffusion. These results support a strong tissue-specificity for each MR sequence.

As indicated above, the cortical layer of the *Drosophila* brain contains mostly the cell bodies of neurons and glia. We visualized all the neuronal cell bodies with the pan-neural nuclear marker Elav (Fig. 7d, arrowheads) and the light blue stain in the brain sections (Fig. 7e, arrowheads). These neurons project both dendrites and axons towards the brain and optic lobe neuropils, where they form multiple highly organized microdomains. In 3D FLASH, the cortical areas showed hyperintense signal compared to the underlying neuropil (Fig. 7f, arrowheads). Conversely, DWI detected low signal from the brain cortex separating the central brain and the optic lobes in several consecutive images (Fig. 7g, arrowheads). Overall, comparing the FLASH and DWI from the same specimen underscored the territory-specific signal by these two MRM contrast modalities, thus revealing relevant structural anisotropies in the sample.

### Dissection of neuropil microarchitecture by DWI

Although DWI demonstrated weak sensitivity to detect retina, lamina, and head muscles, it resolved the organization of the synaptic neuropils with high contrast (Fig. 8). Next, we validated the identity of neuropil microdomains with fluorescent imaging and histological sections in three comparable planes. The mushroom bodies are arguably the best characterized brain centers in *Drosophila* for their role in olfactory memory and higher order processing of complex behaviors^30^. The mushroom body neurons form two clusters of ~2,500 neurons in the posterior brain that send their axons anteriorly in two bundles (the peduncles) that fasciculate into dorsal (α, α′) and medial (β,β′, and γ) lobes (Fig. 8a and b, orange arrow)^31^. At 30 to 50 μm deep, DWI detected the dorsal mushroom body lobes on both sides of the brain as hyperintense signal surrounded by a hypointense halo (Fig. 8c, orange arrow). Since the α/α′ lobes measure 10–15 μm in diameter, DWI can detect anatomic structures close to the voxel size (10 μm^3^). In the same image, the incipient organization of the central complex was appreciated in the center of the brain (Fig. 8a and b, red arrowhead). More ventrally, the suboesophageal ganglion was hyperintense (Fig. 8c, double white arrowhead), while the esophagus produced a large hypointense gap similar to the fluorescent brain (Figs. 8b and c, white arrow). In the histological section though, the esophageal cavity displayed only a small opening corresponding to the lumen of the esophagus, while the rest of the space was filled with the esophageal tube ventrally and the two small muscles of the pulsatile organ dorsally (Fig. 8a, white arrow). Interestingly, DWI detected no signal from the esophagus and the muscles, further supporting the ability of DWI to recognize signal emanating specifically from the brain neuropil.

Moving deeper into the brain (80 μm depth), the axonal tracks of the mushroom body neurons - the peduncles – changed their orientation 90 degrees and became perpendicular to the viewing plane. These thick axonal bundles were prominently labeled in both fluorescence and histological sections (Fig. 8d and E, white arrowhead). In DWI, the peduncles appeared hypointense (Fig. 8f), even though their axonal terminals were hyperintense (Fig. 8c). This low signal was symmetrical and could be followed in several images of the 3D series, supporting the identity of the peduncles. In these same microimages, the central complex formed a green ring in the fluorescent image and a dark blue ring in the histological section, indicating its high synaptic density (Fig. 8d and e, red arrowhead). In DWI, the central complex appeared as a hyperintense ring above the esophageal opening surrounded by a hypointense halo (Fig. 8f). DWI demonstrated high sensitivity for the central complex because the center of the ring was hypointense, appearing very similar to the fluorescent image (Fig. 8d and f).

The third set of images selected corresponded to a depth of 120 μm (Fig. 8g–i). These images contained the hypointense peduncles on the left side due to a slight tilt of the head (Fig. 8i, white arrowhead). Dorsal and lateral to the peduncles was the lateral horn, another higher processing brain center that produced strong GFP signal and blue staining in the histological section (Fig. 8g and h, white diamond). In DWI, the lateral horn also appeared as slightly hyperintense, consistent with other observations that DWI produced higher signal in dense synaptic domains (Fig. 8i). At this depth, we observed the three subdomains of the optic lobes in the same optical sections (Fig. 8g and h): the outer medulla layer (red arrow), the inner lobula layer (rainbow arrowhead), and the lobula plate in between (yellow arrowhead). These optic lobe microdomains appeared strongly stained in the histological sections, but were separated by less dense axonal tracts connecting the synaptic domains (Fig. 8g). In DWI, we detected hyperintense signal from the medulla, lobula, and lobula plate separated by hypointense signal corresponding to the connecting axonal tracts (Fig. 8i). This pattern was symmetrical and could be easily appreciated on the right optic lobe. Finally, at this depth, DWI revealed strong and highly localized signal outside the brain, mostly ventral, corresponding with fat bodies. Overall, DWI seemed to generate stronger signal in the synapse-rich neuropil domains and lower signal in regions with abundant axonal tracts and cell bodies.

## Discussion

Over the last few years, MRM has advanced its resolving power and sensitivity mainly due to the development of dedicated hardware, in particular higher magnetic fields, strong/fast-switching gradient coils, and optimized RF microcoils. Whereas the sensitivity and resolution of MR are two to three orders of magnitude lower than light microscopy, its unique ability to image soft internal tissues non-invasively makes MR a powerful tool with both basic research and clinical applications. To the best of our knowledge, we present here the highest MR resolution for a complete animal head. MRM at 10 μm^3^ resolution revealed the internal anatomy of the *Drosophila* head non-invasively with unprecedented detail for the first time, despite the opaque exoskeleton covering it. Since MR generates endogenous contrast based on water content and tissue composition, MRM eliminates the mechanical perturbations associated with brain dissection. In fact, we noticed that the MRM brain was slightly smaller than the dissected brain and the sectioned head, further supporting the idea that MRM preserves better the spatial arrangement of brain microdomains.

It is well established that T1-weighted imaging with 3D FLASH can reveal morphological features from human and animal brains with a short acquisition time, making FLASH the sequence of choice in two previous publications in living *Drosophila*^22^,^23^. These reports described large internal body structures in some detail, like flight muscles and guts, but lacked the sensitivity to detect internal head structures. Using state-of-the-art hardware and two complementary MR sequences, we detected several internal structures, including central brain, optic lobes, retina, lamina, head muscles, and fat bodies. Although FLASH identified the cortex by hyperintense signal, it provided little detail of the complex organization of the neuropil. To obtain more details of the neuropil microarchitecture, we imaged the same *Drosophila* head with a complementary scheme (DWI), which helped visualize different structures. DWI tags the diffusion of water molecules within the confined neuronal architecture and the contrast stems from the population of slow diffusing water molecules that remain within each voxel due to physical barriers such as cell membranes. The strength of the planar gradient (1.1 T/m/A) and its fast switching times using low-inductance coils demonstrated the ability to attain a micron-scale resolution even with high diffusion weighting using a b-value of 2,000 s/mm^2^. 3D DWI provided an optimal balance between contrast and signal-to-noise ratio at 10 μm^3^, resulting in unprecedented detail of the modular microarchitecture of the brain neuropil. Remarkably, the highest signal in DWI emanated from synapse-rich domains, including the mushroom body lobes, the antennal lobes, the central complex, and the three subdomains of the optic lobes. On the other hand, neuronal cell bodies, axonal tracks, and other structures closely associated to the brain (lamina, retina, esophagus) were hypointense. The resulting 3D reconstruction of the *Drosophila* brain from combining FLASH and DWI revealed the spatial distribution of several known neural microdomains using non-invasive imaging for the first time, suggesting that future applications in basic research may be possible.

This study provided new and relevant observations that may contribute to better interpret how MR extracts signal from complex tissues. First, T1-weighted imaging with FLASH was hyperintense in the retina, the lamina, and the cortical region of the brain corresponding to the position of the cell bodies of neurons and glia (Fig. 6F). Conversely, these three regions were hypointense in DWI, resulting in a complementary pattern between the two imaging protocols. The low DWI signal indicates higher water diffusion rate in these three domains. In principle, cell bodies provide a weaker barrier for water diffusion than the smaller cellular processes of the neuropil, which contains vast amounts of small axons, dendrites, and synaptic terminals. The high diffusion of water molecules in regions of the *Drosophila* brain containing neuronal cell bodies agrees with similar observations in excised rat, porcine, and human spinal cord tissue^9^,^10^. Thus, imaging the same head by two MR modalities revealed complementary sensitivity in several brain regions characterized by FLASH hyperintense and DWI hypointense signals.

The second interesting observation from our study was a DWI signal inversion between positive mushroom body projections and negative peduncles. The peduncles are the forward projecting axons of the Kenyon cells, two large neuronal clusters in the posterior head with a key role in olfactory memory^32^. When the peduncles reach the anterior brain, they branch into medial and dorsal axonal terminals (lobes) that synapse with a large variety of neurons^31^. Two hypotheses can explain this inversion in DWI signal in the same neurons. One possibility is that the orientation of the nerve fibers plays a role in the DWI signal: mushroom body lobes are perpendicular to the diffusion gradient direction, whereas the peduncles are parallel. In that regard, both structures share features in MR signal with myelinated axons in mammals, where the orientation of fatty myelin membranes and microstructures within axons affect the diffusion anisotropy^33^. The second hypothesis is that DWI signal is describing the cellular architecture and membrane density of each domain. The mushroom body terminals form thousands of tiny synapses, whose small processes restrict water diffusion, resulting in strong DWI signal. In contrast, the peduncles are bundles of axons that favor water diffusion along their axes, thus producing low DWI signal. The advantage of DWI for tracing the random movement of water molecules allowed the visualization of synapse-rich structures: when the *b*-value of the diffusion gradient increases above 1,000 s/mm^2^, the diffusion characteristics of water molecules reflect fast moving (hypointense) versus slow moving (hyperintense) regions. Although DWI is highly sensitive for detecting abnormal liquid retention in the brain following ischemic stroke or edema, at microscopic resolution, DWI seems highly sensitive for mapping synaptic domains due to the small cellular processes that restrict water diffusion. This new feature may be particularly useful for developing structural maps and detecting pathological conditions in larger mammalian brains.

The main limitation of our studies is the long acquisition time, around 40 h for each sequence, which is not practical for developing laboratory applications of MR technology. Since in 10 μm^3^ voxels the number of water molecules that can generate MR signal is very small, the only way to produce bright signal by “water imaging” is to increase the scan time. This situation is comparable to optical imaging with poor illumination, which requires longer exposures to create visual signal. Our results provide proof-of-concept that DWI can reveal details of the brain microarchitecture at 10 μm^3^, but we had to use fixed brains to take advantage of a small and sensitive RF coil. Moving to higher magnetic fields and improvements in other hardware components can increase the signal enough to significantly reduce scan times at high resolutions. These advances will enable acquiring DWI data in more directions to build tractography maps for connectomics studies or add additional b-values to construct diffusion maps. Shorter scan times would also allow us to create probabilistic maps of normal and abnormal brains, and to image living samples. But pushing the resolution of MRM to reveal anatomic details at the cellular level will always require making compromises in other parameters, such as using longer scan times and fixed samples.

In summary, a unique collection of dedicated MR hardware allowed us to scan a whole *Drosophila* head non-invasively at an unprecedented resolution. Further improvements in the resolution and/or contrast of MRM may enable to reach cellular resolution in whole brains. Combined with the recent advances in visualizing single cells at high resolution^9^,^10^, MRM has the potential to dissect the brain wiring and function at the cellular level in the near future. However, the most immediate application of MRM technology is to help interpret the signal produced by different MRI schemes at the cellular/tissue level, which can improve the diagnostic power of MRI.

## Methods

### Preparation of Drosophila heads for MRM

*Drosophila* wild type females were grown at 18°C to maximize their size. 24 h after eclosion, flies were anesthetized in CO_2_, the heads were severed with microscissors, and transferred to PBS by centrifuging at 13,000 rpm in a microcentrifuge for 1 min or until the heads were submerged. Then, PBS was removed and replaced with fixative (4% formaldehyde and 2% glutaraldehyde in PBS). The heads were spun down again and kept at 4°C for at least 24 h. Prior to imaging, we washed the heads in PBS to eliminate interference from free fixative. Seven separate intact *Drosophila* heads were imaged exploring the parameters of the MR scans.

### MRM, microcoils and supporting hardware

**Figure 3:**
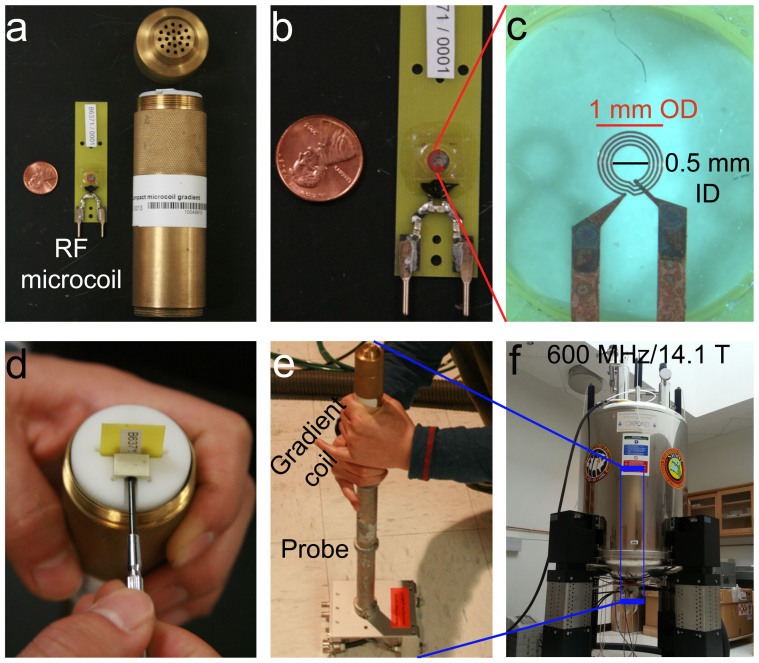
The MRM hardware: magnet, gradient coils, and RF microcoils. (a) The RF planar microcoil and the gradient coil. (b) Detail of the RF microcoil showing the position of the sample well. (c) Magnification of the sample well with the planar 500 μm diameter microcoil. (d) Assembly of the RF microcoil inside the gradient coil. (e) and (f) Assembly of the gradient coil into the probe that is inserted vertically into the center of the magnet.

**Figure 4:**
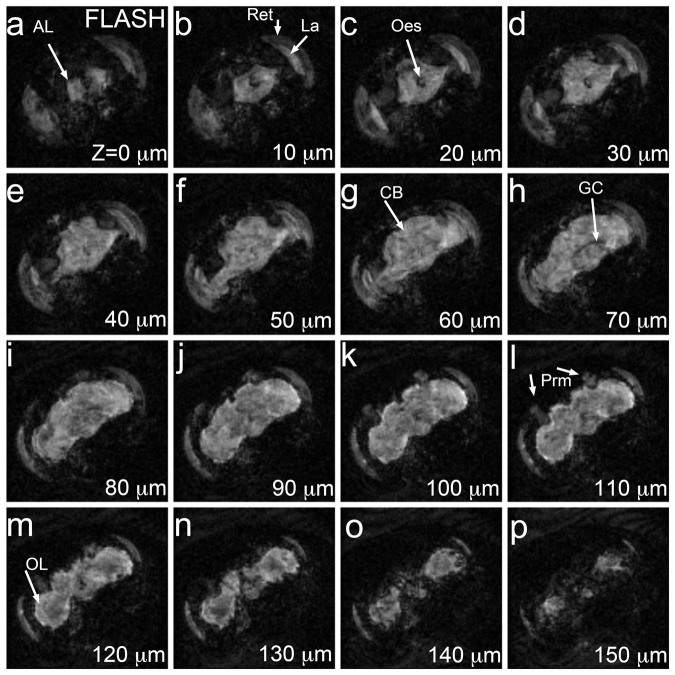
3D FLASH at 10 μm^3^ resolution. 3D FLASH along the frontal plane of a fixed *Drosophila* head. Depth is indicated in μm, starting with the most anterior image. Most internal head structures, including retina, lamina, head muscles, and the entire brain, are hyperintense. From 80 to 130 μm deep, the brain shows a brighter halo in the periphery corresponding to the cortex. Acquisition time was 36 hours. Structures were minimally annotated in these panels to preserve the integrity of the MR images (see figure 2 for abbreviations). CB: central brain; OL: optic lobe. The orientation of these brains is the same as in Fig. 2Aa.

**Figure 5:**
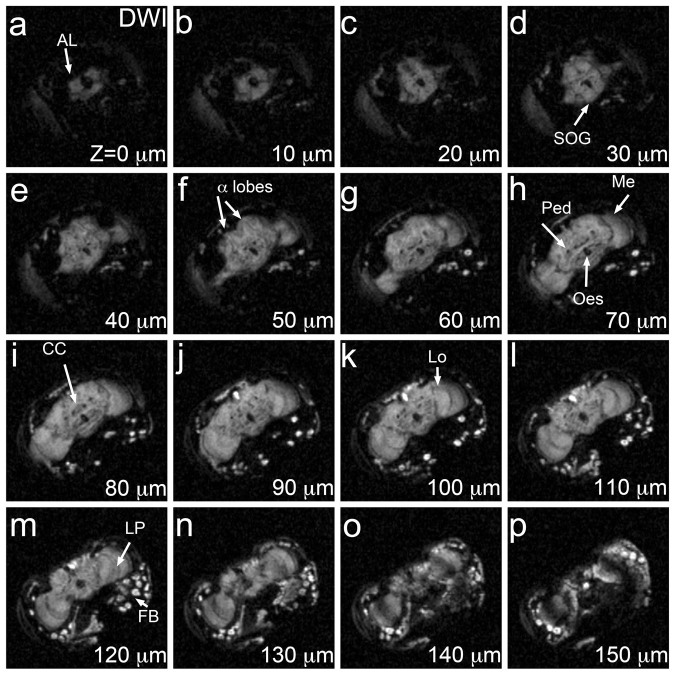
3D DWI at 10 μm^3^ resolution. 3D DW MRM of the same *Drosophila* head. The DW MRM series is slightly darker than FLASH MRM, but provides exquisite contrast in the brain neuropil. Most head structures show low signal, including the retina, the lamina, and the head muscles. But the brain shows fine detail of the modular microarchitecture of the neuropil. Fat bodies in the posterior ventral area appear very bright. Acquisition time was 44 hours. Structures were minimally annotated in these panels to preserve the integrity of the MR images (see figure 2 for abbreviations). The orientation of these brains is the same as in Fig. 2Aa.

**Figure 6:**
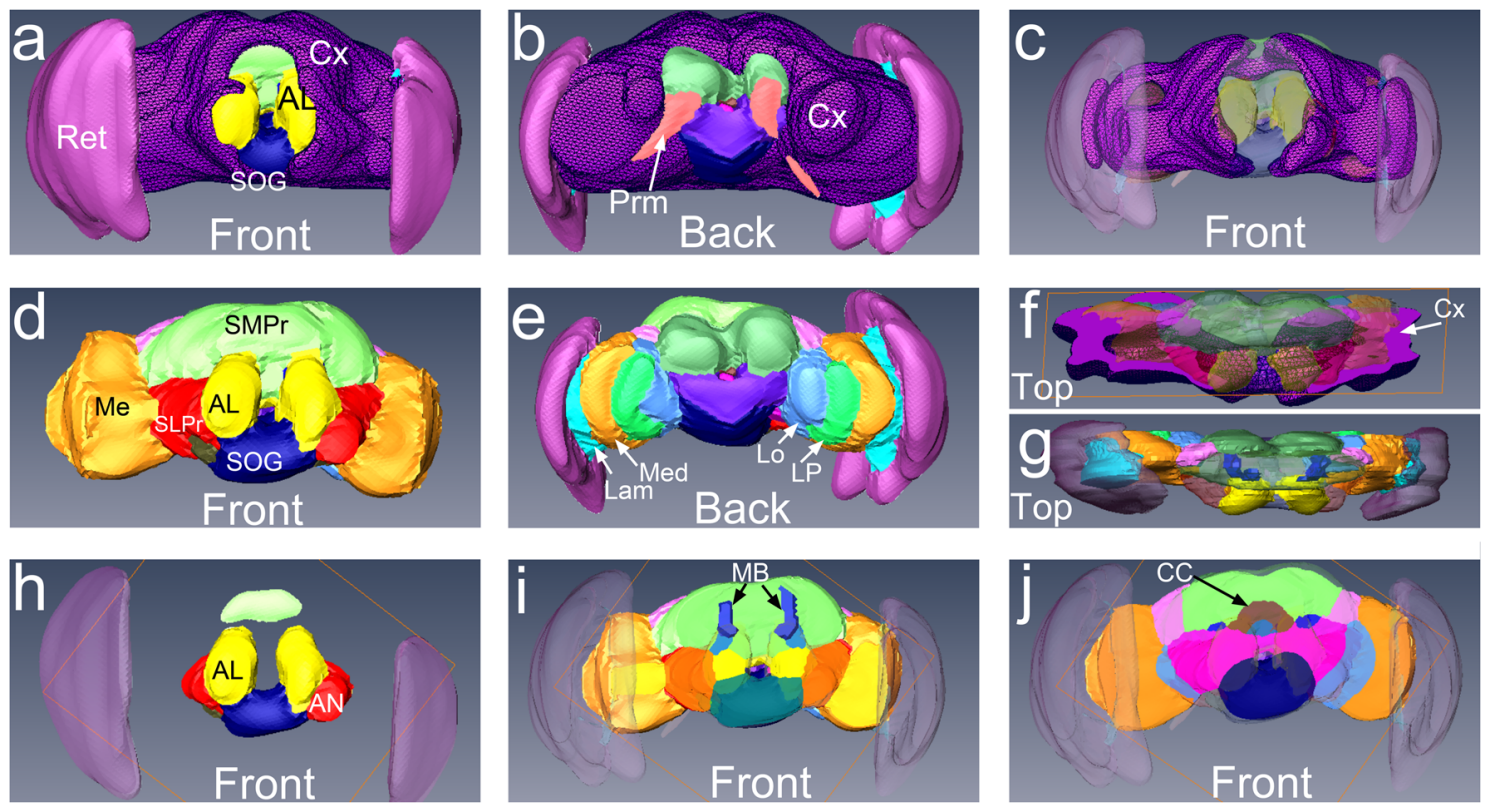
A reconstruction of the 3D architecture of the Drosophila brain. (a) Frontal view of the brain and the eyes. The cell bodies of the cortex are indicated in patterned purple. (b) Posterior view of the brain and the eyes. (c) Frontal view of the cortex with transparent neuropil and retina. (d) Frontal view of the brain neuropil and the eyes. The antennal lobes (yellow) are the anterior most neuropil of the fly brain. (e) Posterior view of the brain neuropil. The different domains of the eye and optic lobes are clearly delineated: retina (purple), lamina (cyan), medulla (orange), lobula plate (green), and lobula (blue). (f) Top view of cortex and neuropil. (g) Top view of the neuropil and eyes. The axonal projections of the mushroom bodies (dark blue) are prominently labeled in the central brain. (h) Simplified frontal view of the brain neuropil showing the antenna lobes (yellow), antennal nerves (red), and suboesophageal ganglion (dark blue). (i) Frontal view of the brain neuropil without the anterior domains. The dorsal projections of the mushroom bodies are indicated in blue, the superior medial protocerebrum in green, and the ventro-lateral protocerebrum in red. (j) Located centrally, we identified the round central complex (light blue and brown). To the sides, we outlined the ventro-medial protocerebrum (pink), to the back the superior-posterior protocerebrum (light green), and lateral to it, the lateral horn (light pink).

**Figure 7:**
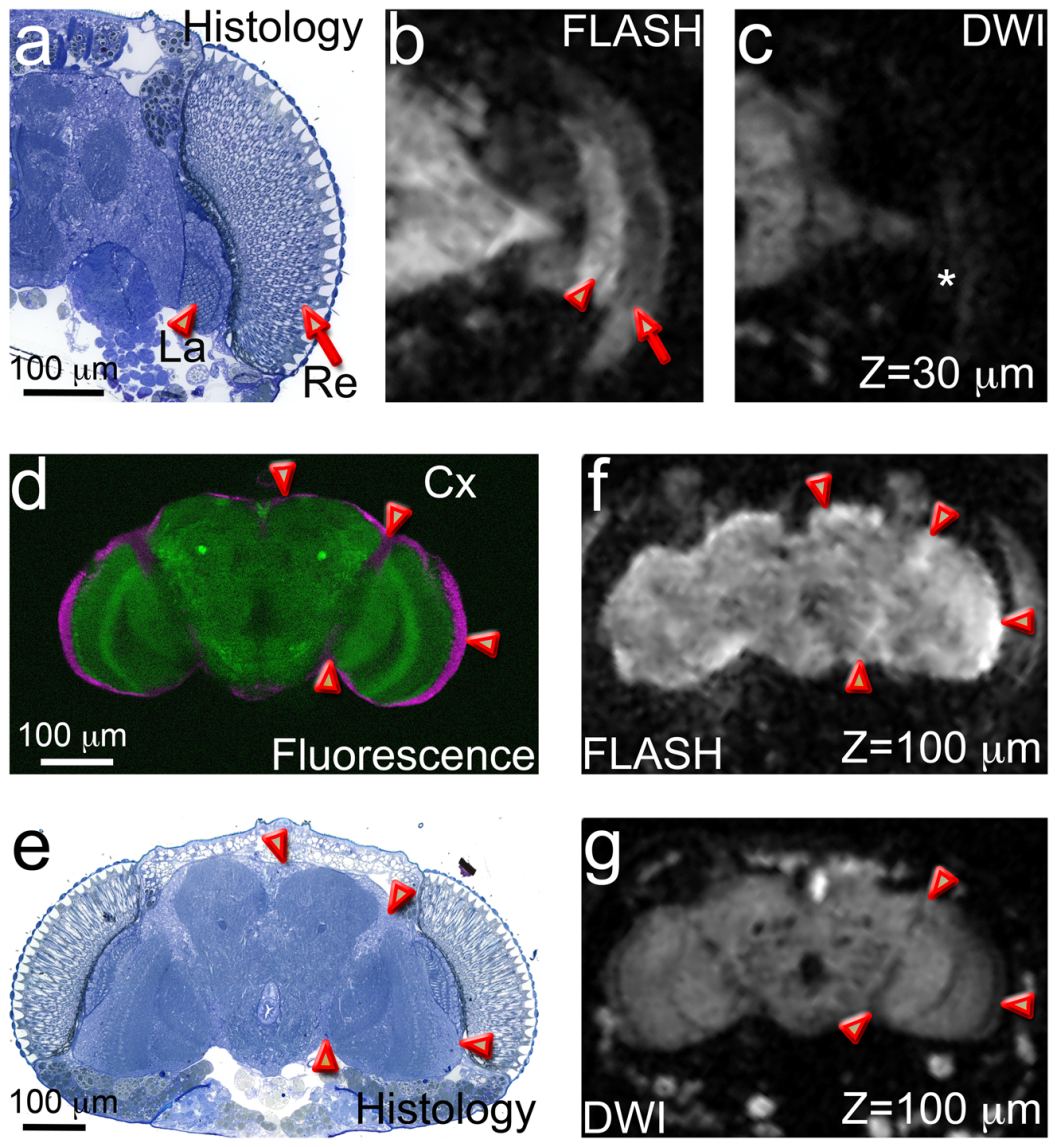
Example of FLASH and DWI microimages discriminating different head structures. (a–c) MRM signal in the retina and the lamina. (a) The fly retina (Re, arrow) is composed of 800 ommatidia, the visual units, containing eight photoreceptors each and surrounded by pigment cells and other support cells. The lamina (La, arrowhead) lies underneath the retina and collects the axonal projections of the photoreceptors into cartridges. (b) In FLASH, the lamina is hyperintense (arrowhead) and the retina produces a weaker signal (arrow). (c) In DWI, both retina and lamina (*) produce weak signal. (d–g) MRM signal in the cortex. (d) In the fluorescent brain, anti-Elav labels the neuronal nuclei in the brain cortex (magenta, arrowheads). (e) In the brain section, the staining is weaker around the central brain and the optic lobes (arrowheads). (f) Single FLASH microimage at 100 μm depth showing stronger signal in the brain periphery (arrowheads) and between the central brain and the optic lobes, where the cell bodies are located. (g) Single DWI microimage at 100 μm depth showing high contrast inside the brain, with hypointense areas between the central brain and the optic lobes, where the cell bodies are located (arrowheads).

**Figure 8:**
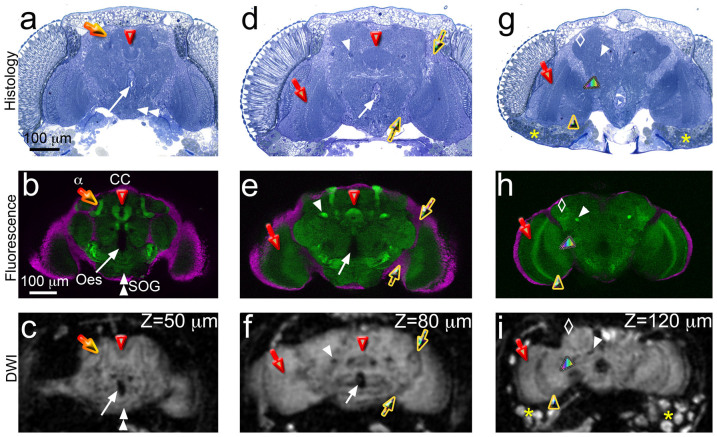
Dissection of the brain neuropil by 3D DWI. (a–c) The anterior-medial brain. (a) and (b) The most prominent structures are the mushroom body lobes (α lobes shown, orange arrow) and the central complex (CC, red arrowhead). The suboesophageal ganglion occupies the ventral side (SOG, double white arrowhead). In the center, the esophageal opening (white arrow) is not labeled in the fluorescent brain, but the histological section reveals the esophagus lining and two small muscles. (c) In DW MRM, the α lobes are hyperintense and are surrounded by a dark halo. The CC is only beginning to show. The SOG is bright ventrally, while the esophageal opening is hypointense. (d–f) The middle brain. (d) and (e) The pedunculus (ped, white arrowhead) forms a thick nerve bundle that crosses the brain, while the CC forms a prominent ring structure in the center. The medulla (red arrow) is the anterior most neuropil of the optic lobes. (f) In DWI, the pedunculus is hypointense, while the CC is surrounded by hypointense tissue. The medulla appears as a homogenous domain of the optic lobes. The esophagus and the cortical area between the central brain and the optic lobes (yellow arrows) are hypointense. (g–i) The posterior brain. (g) and (h) The three domains of the optic lobes are visualized: medulla, lobula (rainbow arrow), and lobula plate (yellow arrowhead). The pedunculus still traverses the brain until it reached the Kenyon cells. The lateral horn (white diamond) shows intense blue and GFP labeling. The fat bodies (yellow *) are indicated in G. (i) In DWI, the three domains of the optic lobes are separated by hypointense axonal tracks. Only one pedunculus is detected due to a slight tilting of the brain. The lateral horn is hyperintense on the left side. The fat bodies produce strong signal ventral to the brain.

MRM was carried out in a 600 MHz (14.1 T) vertical-bore magnet (Oxford Instruments) (Fig. 3f) interfaced with Bruker Biospin console. Strong gradients were provided by a newly designed planar gradient system (Bruker Biospin, Z110828, B6406), capable of 1.1 T/m/A (Fig. 3a). Although these gradients can switch in close to 50 microseconds, the switching time was limited to 100 microseconds in these studies to reduce the stress on the gradients for long 3D scans. The 1 mm diameter micro surface-coil or micro RF probe (Bruker Biospin, B6370/0001) had the optimal size to fit the *Drosophila* head (see Fig. 1c). The fixed heads were placed in the sample well filled with PBS to avoid air bubbles. To restrain the sample close to the coil surface, we wrapped the head with micro-wound 50 μm nylon mesh (Small Parts, Inc.) with a see-through window to fit the head while providing pores for the medium to flow through. Additionally, a polyethylene retention ring with high visibility and biocompatibility was engineered to retain and stabilize the sample and nylon mesh to the surface of the micro coil. An opening was used for irrigating the sample and as a venting hole for unwanted air bubbles. The sample was sealed with adhesive PCR film (ABgene) and covered with and a plastic retention ring engineered for this purpose. All experiments were performed in the Advanced Magnetic Resonance Imaging and Spectroscopy (AMRIS) facility at the McKnight Brain Institute (University of Florida). Scanning parameters: 3D FLASH MRM (TR/TE = 500 ms/15 ms, res = 10 μm^3^, temp = 23°C, Avg = 52, scan time = 36 h) and 3D DW-MRM (TR/TE = 2000 ms/4.7 ms, res = 10 μm^3^, temp = 23°C, Δ = 2.08 ms, δ = 0.19 ms, b = 2000 s/mm^2^, Avg = 16, scan time = 44 h) were collected. The diffusion sensitization gradient was aligned with the direction of slice-selection gradient axis. Only one b-value (high) was acquired to minimize the acquisition times of combining FLASH and DWI scans.

### Immunofluorescence

For the fluorescent brain, we generated flies expressing GFP (green fluorescent protein) localized to the synapse in all neurons (Elav-Gal4; UAS-Synaptobrevin (Syb)-GFP^34^. Adult females were collected 24 h after eclosion and the brains were dissected and fixed in 4% formaldehyde. Then, brains were incubated with the anti-Elav antibody (1:50, Developmental Studies Hybridoma Bank) to label the nuclei of all the neurons followed by secondary anti-rat Cy3 antibody (1:600; Sigma). The stained brains were mounted in Vectashield (Vector Labs) with two glass supports to prevent squeezing and imaged in an Axio Observed Z1 Apotome Zeiss microscope by structured light imaging with a plan-apochromat 10× objective (NA-0.45) using the Multicolor module of AxioVision (Zeiss). Complete brains were collected as stacks with a z-step of 0.7 μm and representative images are shown as single optical planes.

### Histological sections

The heads from wild type females were severed with microscissors 24 h after eclosion, transferred to fixative (glutaraldehyde 3%) by centrifuging at 13,000 rpm for 1 min or until the heads sink, and incubated for 24 h at 4°C. The heads were then washed in phosphate buffer and post-fixed in OsO_4_ for 1 h, serially dehydrated, and infiltrated with Epon overnight as described before^35^. 1 μm semi-thin sections were obtained in a Leica microtome with a diamond knife, stained for 5 min with toluidine blue, washed, air dried, and mounted in DPX (Sigma). Representative sections were imaged in an Axio Observer Z1 Zeiss microscope with a plan-apochromat 20× objective (NA-0.8) using the MosaiX module in AxioVision (Zeiss) by stitching four partially overlapping images.

### Other imaging techniques, 3D rendering and image processing

We imaged the adult head on the coil with a Leica Z16 APO using a 2× plan-apochromat objective, collected a z-stack, and generated a single in-focus projection with the Montage Multifocus module of the Leica Application Software. To generate the 3D model of the *Drosophila* brain, we opened the 3D FLASH MRM and 3D DW MRM data sets in Amira (version 5.4.0, Visage Imaging Inc) and segmented internal head structures manually. Figures were created in Photoshop and images received minimum manipulation to adjust intensity and contrast to whole images.
